# The Role of Plant–Microbe Interactions and Their Exploitation for Phytoremediation of Air Pollutants

**DOI:** 10.3390/ijms161025576

**Published:** 2015-10-26

**Authors:** Nele Weyens, Sofie Thijs, Robert Popek, Nele Witters, Arkadiusz Przybysz, Jordan Espenshade, Helena Gawronska, Jaco Vangronsveld, Stanislaw W. Gawronski

**Affiliations:** 1 Centre for Environmental Sciences, Hasselt University, Agoralaan building D, Diepenbeek 3590, Belgium; E-Mails: sofie.thijs@uhasselt.be (S.T.); nele.witters@uhasselt.be (N.W.); jordan.espenshade@uhasselt.be (J.E.); jaco.vangronsveld@uhasselt.be (J.V.); 2 Faculty of Horticulture, Biotechnology and Landscape Architecture, Warsaw University of Life Sciences, Nowoursynowska 159, Warsaw 02-766, Poland; E-Mails: robert.popek@gmail.com (R.P.); arek.przybysz@gmail.com (A.P.); helena_gawronska@sggw.pl (H.G.); stanislaw_gawronski@sggw.pl (S.W.G.)

**Keywords:** phytoremediation, air pollutants, phylloremediation, particulate matter, VOCs (volatile organic compounds), microbiome

## Abstract

Since air pollution has been linked to a plethora of human health problems, strategies to improve air quality are indispensable. Despite the complexity in composition of air pollution, phytoremediation was shown to be effective in cleaning air. Plants are known to scavenge significant amounts of air pollutants on their aboveground plant parts. Leaf fall and runoff lead to transfer of (part of) the adsorbed pollutants to the soil and rhizosphere below. After uptake in the roots and leaves, plants can metabolize, sequestrate and/or excrete air pollutants. In addition, plant-associated microorganisms play an important role by degrading, detoxifying or sequestrating the pollutants and by promoting plant growth. In this review, an overview of the available knowledge about the role and potential of plant–microbe interactions to improve indoor and outdoor air quality is provided. Most importantly, common air pollutants (particulate matter, volatile organic compounds and inorganic air pollutants) and their toxicity are described. For each of these pollutant types, a concise overview of the specific contributions of the plant and its microbiome is presented. To conclude, the state of the art and its related future challenges are presented.

## 1. Introduction

Air pollution has become a major cause of concern worldwide. The origin of airborne pollutants is often related to thermal processes (e.g., combustion of fuels). A lot of epidemiologic research has disclosed associations between air pollution and adverse health effects [1,2,3,4,5]. Moreover, data from 20 United States (US) cities showed that levels of PM_10_ (PM ≤ 10 μm) can be correlated with higher mortality rates as a result of cardiovascular or respiratory disorders [6] and, in recent years, it was recognized that exposure to PM during pregnancy or early life may be linked to developing autism spectrum disorder (ASD) [7,8]. Current emission abatement strategies, focusing on specific technical measures, are not sufficient to meet either environmental or climate challenges. Although improvements in combustion technology are likely to reduce the overall emissions, and subsequent exposure, highly populated areas continue to be severely challenged by high emissions. Despite all efforts, the last annual report on air quality in Europe estimated that many urban inhabitants in the EU are still exposed to air pollutant concentrations above the World Health Organization (WHO) guidelines (Table 1) [9].

**Table 1:** World Health Organization (WHO) guidelines (2006) for most important, monitored air pollutants.

Pollutant	Averaging Period	Max Number of Exceedances	WHO Guideline
PM_10_	1 day	3	50 μg/m^3^
	1 year	NA	20 μg/m^3^
PM_2.5_	1 day	3	25 μg/m^3^
	1 year	NA	10 μg/m^3^
Ozone	Max daily 8 h	0	100 μg/m^3^
NO_x_	1 h	0	200 μg/m^3^
	1 year	NA	40 μg/m^3^
SO_x_	10 min	NA	500 μg/m^3^
	1 day	0	20 μg/m^3^

PM_10_: fraction of particulate matter with an aerodynamic diameter less than 10 μm; PM_2.5_: fraction of particulate matter with an aerodynamic diameter less than 2.5 μm; NA: data not available.

Ambient air pollution is composed of a high variety of primary and secondary pollutants, mainly including particulate matter (PM), volatile organic compounds (VOCs) (benzene, toluene, ethylbenzene, xylene (BTEX), poly aromatic hydrocarbons (PAHs), formaldehyde, and so on) and inorganic pollutants (NO_x_, SO_2_, CO_2_, O_3_). Many of these outdoor air pollutants are also found indoor, in concentrations that often can be higher than the outdoors [10].

Despite the complexity in composition, phytoremediation was already shown to be an effective plant-based, environmentally friendly biotechnology to reduce and detoxify/degrade indoor and outdoor air pollutants. Plants are known to scavenge significant amounts of air pollutants and even partly metabolize them [11,12]. Fortunately, plants do not live alone; they are known to be associated with thousands if not millions of other organisms, such as fungi and bacteria. The functions of these plant-associated microorganisms are still under investigation, but they are well known to support plants to cope with abiotic and biotic stresses, to assist their host in nutrient and water uptake, and to produce plant hormones, siderophores and inhibitory allelochemicals [13,14,15]. In general, it is recognized that plant–microbe interactions play an important role during phytoremediation by degrading, detoxifying or sequestrating the pollutants and by promoting plant growth [15,16]. Some research showed that growing plants indoor increases air humidity, but contrarily to using industrially produced devices, it is not accompanied by an increase of harmful (for humans), colony forming units (cfu). Most probably, this is due to allelochemicals that are released to the atmosphere by the plants’ microbiome and that are inhibiting growth of airborne microorganisms [17,18].

**Figure 1:**
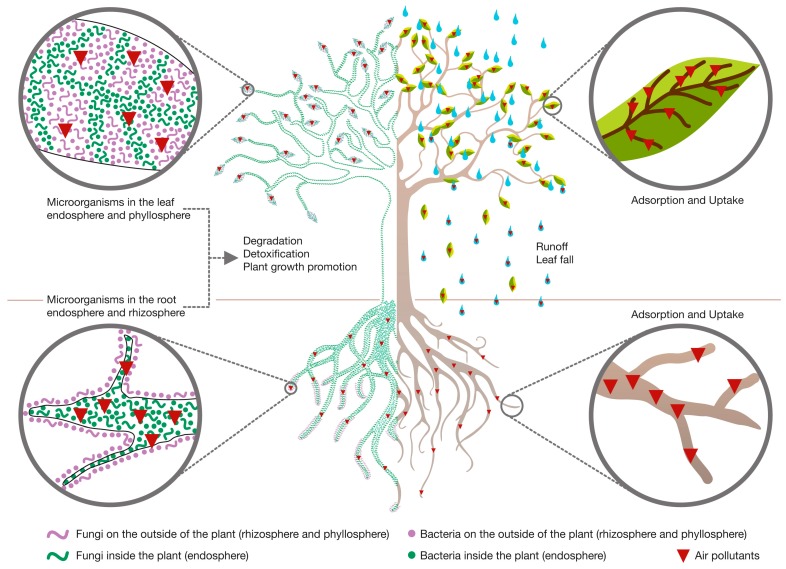
Schematic overview of phytoremediation of air pollution.

In case of air pollution, the surface of leaves and stems is known to adsorb significant amounts of pollutants. Therefore, bacteria living on these surfaces, called the phyllosphere bacteria, might be of high importance. Part of the adsorbed pollution is also finding its entry into the plant, making (especially) leaf endophytes of high interest. These phyllospheric and endophytic bacteria can detoxify part of the pollutants by means of degradation, transformation or sequestration. Further, rainfall causes flowing down of the pollutants to the soil right below the plant, where the pollutants come into contact with the soil, the plant’s rhizosphere and the roots. A schematic overview of phytoremediation of air pollutants is presented in Figure 1.

**Figure 2:**
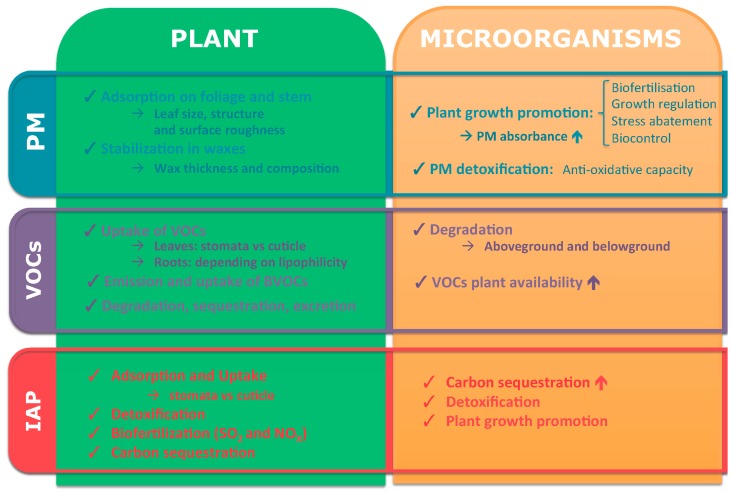
A concise overview of the specific contributions of the plant and its microbiome to the phytoremediation of the different categories of air pollution (increasing effects are indicated with 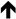).

In this review, the available knowledge about the above-described plant–microbe interactions during phytoremediation of air pollution is summarized for the main air pollutants (particulate matter, volatile organic compounds and inorganic pollutants). For each of these pollution categories, a definition, the toxicity and the role of the plants and their associated microorganisms is described. Moreover a concise overview of the specific contributions of the plant and its microbiome is presented in Figure 2. To conclude, the state of the art and its related future challenges are provided.

## 2. Particulate Matter

### 2.1. Definition and (Human) Toxicity

Particulate matter (PM) is a mixture of solid and liquid substances with different origins, shapes and chemical compositions [19].

PM can be generated (outdoors) by human activity, for example, vehicle exhausts, road dust, fossil fuels, and industrial activities [20]. Moreover, PM is also generated indoors, mainly by heating, cleaning and cooking activities [10,20,21,22,23]. Next to these anthropogenic emission sources, significant amounts of PM can also be generated naturally by e.g., volcanic eruptions, forest and prairie fires, sandstorms, ocean breezes and soil and rock erosion.

Mostly, PM is classified in four fractions based on its aerodynamic diameter (Ø): large (Ø: 10–100 μm), coarse (Ø: 2.5–10 μm), fine (Ø: 0.01–2.5 μm) and ultrafine (Ø: < 0.01 μm) PM [24].

Particulate matter is composed of a relatively non-reactive part, as for example carbon or calcium, to which biologically active chemicals like (toxic) metals, organic compounds (e.g., PAHs) [25] and environmentally persistent free radicals (EPFRs) can be adsorbed [26], making them even more toxic.

Particulate matter is widely recognized as one of the most dangerous pollutants for human health [27,28,29,30,31,32]. Fine and super fine (PM_2.5_) particulate matter alone are causing over 2 million deaths on an annual basis all over the world [33]. Due to the highly variable chemical and physical composition of PM, toxicological studies have not succeeded in determining the exact mechanisms of PM-induced toxicity so far. Many studies indicate that the level of PM toxicity is related to the chemical composition, particle size and shape [34]. The most dominant hypothesis is that ultrafine particles (UFP) are more toxic compared to fine and coarse PM (Table 1) and, further, that toxicity is caused by inducing the generation of reactive oxygen species (ROS) on their surface. Only recently, Kiruri *et al.* [35], Kelley *et al.* [36] and Khachatryan *et al.* [37] showed that this ROS production on the surface of UFP is related to surface-associated environmentally persistent free radicals (EPFRs).

### 2.2. Role of Plants during PM Phytoremediation

Plants are known to be capable of scavenging significant amounts of PM, especially in urban areas and close to roads, by adsorbing PM on the foliage (_s_PM) or stabilizing them in waxes (_w_PM) [38,39,40,41,42]. Popek *et al.* [43] demonstrated that trees and shrubs, creating a biofilter on a way of PM flow, reduced the amount of PM that is accumulated on the foliage of trees grown further away in the park by about 50%. Both modeling and experimental (laboratory) research have been performed on PM scavenging by urban greenery around the world. The most used model to describe the urban forest structure and its ecosystem services, such as pollutant removal, is the i-Tree model developed by Nowak *et al.* [44].

For example, it was estimated that in Beijing (China) trees in the city center removed 772 tons of PM_10_ on a yearly basis [45]. In Shanghai (China), a 9.1% decrease in PM concentrations was observed at a distance of 50–100 m into a forest in comparison with external urban woodland [46]. McDonald *et al.* [47] showed that planting trees in the West Midlands (UK) on 3.7% up to 54% of the available land would reduce PM_10_ concentrations in the air by 26%, causing the removal of about 200 tons of PM_10_ per year. In Chicago, USA, trees occupying 11% of the city area eliminated approximately 234 tons of PM_10_ per year [48] and in the USA as a whole, trees and shrubs in urban areas adsorb around 215,000 tons of PM_10_ annually, representing a monetary value of 969 million dollars [11].

Although these numbers are very positive, we should keep in mind that 10–20-fold differences in PM accumulation among plant species were observed [39].

Taking into account their large total leaf area, trees are regarded as the most effective type of vegetation for PM scavenging [47]. Moreover, the architecture of tree crowns resulting from the complex structure of foliage and shoot induces turbulent air movement, which positively affects PM accumulation capacity [49,50]. Next to trees, herbaceous vegetations have also been shown to be effective PM scavengers [51]. The air filtration process can be enhanced by species-specific features of leaves, such as trichomes and the amount, chemical composition as well as the structure of epicuticular waxes. These wax layers are known to be able to immobilize and phytostabilize adsorbed PM [38,40,52]. In summary, plant-specific traits like leaf size and structure, wax content, ultrastructure and thickness, and pubescence and surface roughness, but also climate conditions such as precipitation and wind, and PM quantity and composition can affect the PM scavenging capacity [11,39,53,54,55].

Once PM is accumulated on plant leaves, it might affect their optical properties by absorption/reflection of PAR (photosynthetically active radiation) or clogged stomata resulting in a negative effect on photosynthesis and transpiration [42,56]. Photosynthesis and other physiological processes are also affected by toxic compounds attached to the surface of PM, e.g., trace elements, organic pollutants and Cl^−^ and Na^+^, that, depending on the type and environmental conditions, may penetrate into plant tissues or can be removed from the surface of foliage by rain or wind events [57,58]. Przybysz *et al.* [59] found a negative correlation between photosynthesis rate and the level of accumulated PM, proving that photosynthesis efficiency depends, at least to some extent, on the level of PM. This negative effect on the photosynthetic apparatus was confirmed by a lower chlorophyll content and photosynthesis rate, an increased stomatal resistance and a decrease in the fluorescence of chlorophyll *a* parameters values. Although several other authors found similar negative correlation between PM and photosynthesis rates [56,60,61], for some plant species such as *Ilex rotunda* trees [62] and *Sorbaria sorbifolia* [59], the opposite was observed: photosynthetic rate was in some species higher in the more polluted areas. This is explained by the possible protective role of PM by reducing photoinhibition and probably a better (species-specific) tolerance for the PM-induced oxidative stress.

Overall, it is clear that both the PM accumulation capacity as well as the response of the photosynthetic apparatus are highly plant species specific.

### 2.3. Role of Plant-Associated Microorganisms during PM Phytoremediation

Plant-associated microorganisms are known to play an important role during plant growth and development by increasing nutrient availability (e.g., production of organic acids, siderophores), by producing plant growth hormones (e.g., production of indole acetic acid (IAA)) and by helping the plant to cope with abiotic and biotic stresses (e.g., production of 1-aminocyclopropane-1-carboxylate (ACC) deaminase) [13,14,15].

In case of PM phytoremediation, these plant growth-promoting traits might result in an increased biomass and thus surface to adsorb pollutants, meaning an improved PM adsorbance capacity. In general, direct and indirect mechanisms can induce plant growth promotion, as described by Weyens *et al.* [15].

Direct plant growth promotion can be resumed in three topics, which are further discussed below: bio-fertilization, growth and development regulation and stress abatement. (1) Some of the mineral nutrients, including nitrogen, phosphorus and iron, are frequently limiting in soil, and by consequence inhibiting the growth of land plants. Plant-associated microorganisms can act as bio fertilizers by fixing and/or solubilizing mineral nutrients that are unavailable for plants. Among those processes, biological N_2_ fixation by rhizobia is well-known. Nodulated leguminous plants incorporate C and N into soil, which besides increasing nutrient uptake capacity, also improves their tolerance to environmental stresses [63]. Moreover, Rhizobia have been shown to be a potential tool for the remediation of organic and metal contaminations, by degrading organic contaminants and adsorbing, accumulating and detoxifying [64]. (2) Bacteria are able to produce plant growth regulators such as auxins (e.g., IAA), cytokinins and gibberellins [65]. These phytohormones often can induce a beneficial effect on plant growth and development [66,67]. Interestingly, the production of phytohormones by bacteria does not directly benefit themselves, but indirect benefits are achieved by the increase in nutrient supply, induced by the stimulated plant growth. (3) Negative effects of stress on plant growth can be abated by bacteria through the production of 1-aminocylcopropane-1-carboxylate (ACC) deaminase [68,69]. The general response of plants to (all kinds of) environmental stressors including pollutants is the production of ethylene leading to the activation of processes that inhibit plant development and growth including (but not limited to) senescence, chlorosis and leaf abscission [70]. The ACC-deaminase enzyme, produced by many PGP bacteria, hydrolyzes ACC into ammonia and α-ketobutyrate [71]. As ACC is the immediate precursor for ethylene, lowering the level of ACC in the plant also lowers the amount of ethylene that can be produced. The indirect mechanisms of plant growth promotion can be summarized as the inhibition of the growth and activity of plant pathogens. This inhibition can be induced by various mechanisms including the competition for space and nutrients, the production of biocontrol agents such as antibiotics and antifungal metabolites and/or the induction of systemic resistance [72,73].

Next to their plant growth promoting traits, resulting in higher PM absorbance capacity, plant-associated microorganisms might also play a role in the detoxification of the PM absorbed by their host plant. As described above, PM toxicity is caused by inducing the generation of reactive oxygen species (ROS) on their surface. It is known that some bacteria have high antioxidative properties [74,75], which can play a role in detoxifying ROS. As this ROS production on the surface of ultrafine particles is related to surface-associated EPFRs [35,36,37], we might expect a potential remedial action of bacteria on EPFR by means of (a) a reduction of the EPFR concentration on the surface of PM and (b) a neutralization of ROS species formed by EPFRs in the solution.

Plant-associated microorganisms possess degradation pathways and metabolic capabilities, resulting in more efficient organic contaminant degradation and reduction of both phytotoxicity and evapotranspiration of volatile pollutants [16]. In case of toxic trace elements in the soil, root endophytes equipped with a metal-resistance/sequestration system can decrease metal phytotoxicity and enhance their accumulation in plant tissues [76]. Therefore, it might be expected that foliage-associated microbes may support plants to cope with stresses caused by PM bounded contaminants and enhance phytoremediation efficiency. However, the role of microbes in detoxification of contaminants on the surface of leaves is still poorly understood.

## 3. Volatile Organic Compounds (VOCs)

### 3.1. Definition and (Human) Toxicity

There are numerous definitions to explain “VOC” and, mostly, they are based on physical and chemical features (boiling range, vapour pressure) and/or composition (carbon number range). The basic definition is the one provided by the Solvents Emission Directive: “any organic compound having at 20 °C a vapour pressure of 0.01 kPa or more or having a corresponding volatility under the particular conditions of use” [77]. The presence of VOCs is negatively affecting outdoor as well as indoor air quality. VOCs in the ambient air are mainly of high interest because they significantly contribute to the formation of ozone (O_3_) in the presence of sunlight and nitrogen oxides [78,79]. In case of indoor VOCs, ozone formation is not a problem, since ozone decomposes into oxygen when it comes into contact with any surface (e.g., a wall).

VOCs sources are either anthropogenic (AVOCs) (transport, industry) or biogenic (BVOCs) (trees and other plants). Although on a global scale BVOC fluxes highly exceed that of the AVOC, in urban regions, the large amount of AVOC emissions from industrial and traffic sources results in a relatively low BVOC proportion [80,81]. Indoors, VOCs are emitted from various materials such as carpets, wallpaper, curtains, paper products, office chairs, and electronic equipment with the highest emissions when the material is new [82,83]. In general, the most studied AVOCs are Benzene, Toluene, Ethylbenzene, Xylene (BTEX), Poly Aromatic Hydrocarbons (PAHs), and formaldehyde; and for the BVOCs, chloromethane, isoprene and monoterpenes are most abundant [84].

Next to their role in O_3_ formation, VOCs themselves are also known to induce both short and long term adverse health effects on humans [85,86]. For example, formaldehyde can cause sensory irritation and nasopharyngeal cancer and benzene might lead to blood dyscrasias [87]. As VOCs are the principal pollutants of indoor air [88,89] and people generally spend up to 90% of their time inside buildings (houses, offices, factories, *etc.*), toxicity of VOCs in indoor air are the subject of numerous studies. High indoor levels of VOCs are known to cause multiple chemical sensitivity and the “sick building syndrome” [88,90,91] and a cross-section of physical symptoms (e.g., allergies, asthma and headache) for those who are exposed [86,92].

### 3.2. Role of Plants during VOCs’ Phytoremediation

Several studies have described the ability of plants to remove VOCs from the air [93,94,95,96,97,98]. In a recent review of Dela Cruz *et al.* [99], more than 100 indoor plant species and their VOC removal capacity are summarized in a table. As already mentioned above, it is important to keep in mind that plants can also be an important source of VOCs [100]. Therefore, low VOC emitting plant species should be selected for VOC phytoremediation. More integrative studies already revealed that selecting the optimal tree species composition and a slight increase in tree density result in a substantial (B)VOC reduction and a superior ecosystem service value [100].

In general, plants remove VOCs predominantly by uptake via leaf stomata, yet some gases are removed by the plant surface (cuticle). Uptake through the stomata is confirmed in many studies by a higher removal in light than in darkness (stomata are open in light and closed in darkness) [95,101,102]. Exceptions are so-called CAM and facultative CAM plants, which either constitutively, or after drought stress exposure (facultative) close their stomata during the day and open them during the night [103]. This feature is desired for air phytoremediation because such plants, under drought conditions, take up pollutants from the air during the night, along with their CO_2_ absorption. Many species of *Sedum* genera have the ability of switching to CAM photosynthesis [104], which explains their successful cultivation on extensive green roofs, where drought often occurs. Plants that are recommended for indoor phytoremediation sometimes also experience drought. Species like *Zamioculcas zamiifolia* [105], also a facultative CAM plant, are very efficient for both growth and development as well as uptake of BTEX from indoor air. It is noteworthy that CAM plants grown indoor, besides their air purification traits, are also valuable as they do not compete for oxygen with humans. Facultative CAM systems, when joined with achievements of phytoremediation, are expected to strongly contribute towards our goal in improvement of phytoremediation biotechnologies.

Cuticular absorption was shown by measuring the amount of VOCs present in the wax layer [101,105]. Studies examining the role of both stomata and cuticle uptake by ^14^C labeling concluded a dominant uptake through the stomata and a substantial uptake through the cuticle [106]. Moreover, Dela Cruz *et al.* [99] emphasized the importance of the properties of the VOCs. A hydrophilic VOC will not diffuse easily through the cuticle existing of lipids, whereas a lipophilic VOC is more likely to penetrate through the cuticle. After entering the leaves, VOCs diffuse into intercellular spaces and may be absorbed by water films to form acids or react with inner-leaf surfaces [107]. After uptake in the leaves, VOCs can be translocated through the phloem to various plant organs (e.g., seeds, roots) [108,109].

Part of the VOC air pollutants that are adsorbed by the leaves are moving to the soil below by runoff (by rain) and leaf fall. Here, root adsorbance and uptake come into the picture. Root uptake of organic compounds from soil is affected by (a) the physical and chemical characteristics of the compound; (b) the environmental conditions (e.g., organic matter, pH and moisture); and (c) by plant properties (e.g., root surface area) [110,111]. In case the plant- and environment-related parameters are stable, root uptake is directly proportional to the chemical’s lipophilicity, which can be represented by the chemical’s octanol-water partition coefficient (*K*_ow_). In practice, an optimal range of lipophilicity exists (log *K*_ow_ between 1 and 3.5) outside of which plant uptake and translocation of organics is strongly delimited. Organic contaminants with a log *K*_ow_<1 are known to be highly water-soluble and are lacking any specific affinity to be taken up into plant roots [112], whereas contaminants with a log *K*_ow_>3.5 are so strongly absorbed onto root surfaces that their uptake and translocation to the shoot is limited [113].

Once inside the plant (root or leaf), VOCs can undergo degradation, storage or excretion. For example, formaldehyde can be transformed into 2-C skeletons that can serve as a energy source and be used for biosynthesis of novel molecules [97] and after transformation to CO_2_ it also can be built into the plant material via the Calvin cycle [114]. After ring cleavage, benzene and toluene can also enter the Calvin cycle where they are converted to organic and amino acids [106]. Korte *et al.* [115] reviewed the degradation of xenobiotics in the ambient air. Although degradation to harmless constituents is the optimal goal, storage and excretion are necessary if degradation cannot occur. Moreover, considering VOCs’ degradation, plants are disadvantaged in two ways. Firstly, plants do not rely on organic compounds as a source of energy or carbon since they are phototrophic. By consequence, plants were not under selective pressure to develop the capacity to degrade chemically intransigent materials, which is in contrast with microbial systems. This resulted in a more restricted set of chemicals that can be metabolized for plants, in comparison with micro-organisms. Secondly, plant metabolism of organic carbon (other than photosynthates) follows the green liver model, meaning that first general transformations to more water-soluble forms occur, followed by sequestration processes to avoid build-up and potential toxicity to sensitive organelles [116]. On the contrary, microbial metabolism often results in the compound being transformed to CO_2_, water and cellular biomass. Taking this into account, it is clear that plants rely on their associated microorganisms to obtain a more efficient degradation of VOCs.

### 3.3. Role of Plant-Associated Microorganisms during VOCs’ Phytoremediation

The ability of plant leaves to scavenge VOCs has been well known for a long time, but it is only recently that leaves have been shown to host several VOC-degrading microorganisms. The phyllosphere is one of the most prevalent microbial habitats on earth: the global bacterial population present in the phyllosphere could comprise up to 10^26^ cells [117]; fungal populations are less numerous [118,119,120] and archaea are rather a minor component or even not abundant [121,122]. These phyllosphere communities are strongly affected by a variety of environmental factors, including UV exposure, pollution, nitrogen fertilization, water limitations and high temperature shifts, as well as biotic factors, such as leaf age and the co-presence of other microorganisms [117,123]. As plants themselves produce (B)VOCs in their phyllosphere, the presence of VOC metabolizing microorganisms in the phyllosphere can be expected. However, there are only a limited number of reports that plant leaves accommodate VOC metabolizing microorganisms in their phyllosphere. An overview of the available research on phyllosphere microorganisms in the framework of VOC (including most important AVOCs and BVOCs) phytoremediation is provided in Table 2. These phyllosphere VOC degrading microorganisms are expected to hold great potential in indoor and outdoor air cleanup.

Next to the aboveground plant parts, the belowground plant parts are also highly efficient VOC removers. In this context, the general capability of root-associated microorganisms to metabolize organic compounds has long been established and it has been widely exploited in soil and (ground)water bioremediation programs [16,65,124,125,126,127]. Soil also contains air, of which the amount varies depending on the soil moisture. During drying, the air together with pollutants penetrates the soil and the pollutants are degraded by the more efficient degradation system functioning in soil. After water supply (rain and irrigation), more clean air is forced out into the atmosphere. This phenomenon takes place also in the pots with plants during indoor phytoremediation [128]. Several endophytic and rhizospheric bacteria have been identified as capable of assisting their host in removing toxic compounds from soil [125]. Next to plant-associated bacteria, mycorrhizal fungi have been reported to be equally important for the mineralization of pollutants [129,130,131]. Moreover, several studies have shown that these beneficial, contaminant-degrading actions of microorganisms are enhanced because of the presence of the plant [132,133,134].

In summary, microorganisms associated with the above- and belowground plant parts are important facilitators of phytoremediation of VOCs through their degradation capacity. Moreover, plant-associated microorganisms might also play an important role in enhancing (mainly hydrophobic) VOCs’ bioavailability for the plant via the production of biosurfactants, extracellular polymeric substances or through biofilm formation [135].

**Table 2:** Overview of available research on phyllosphere microorganisms in the framework of VOC (including most important AVOCs and BVOCs) phytoremediation.

Plants	Microbes	VOCs	References
Plant species used for phytoremediation	Bacterial groups with identified role in phytoremediation, predominantly Actinobacteria and Firmicutes	Aromatic and aliphatic hydrocarbons	Al-Awadhi *et al.* [136]
Peas, beans, tomatoes, and squash	*Bacillus, Ochrobactrum, Enterobacter, Rhodococcus, Arthrobacter, Pontola, Nocardia,* and *Pseudoxanthomonas*	*n*-Hexadecane, *n*-decosane, phenanthrene, and crude oil	Al-Awadhi *et al.* [137]
*Halonemum strobilaceum*	*Ochrobactrum* sp and *Desulfovibrio* sp.	Aliphatic and aromatic hydrocarbons	Al-Mailem *et al.* [138]
Bean and maize	*Acinetobacter*, *Alcaligenes*, and *Rhodococcus*.	Phenol	Sandhu *et al.* [139,140]
Ten evergreen ornamental plants	*Acinetobacter*, *Pseudomonas*, *Pseudoxanthomonas*, *Mycobacterium*	Acenaphthylene, acenaphthene, fluorine and phenanthrene	Yutthammo *et al.* [141]
Peas, beans, tomato and sunflower	*Microbacterium* spp., *Rhodococcus* spp., *Citrobacter freundii*	Crude oil, phenanthrene and *n*-octadecane	Ali *et al.* [142]
Sixteen cultivated and wild plant species from Kuwait	*Flavobacterium*, *Halomonas*, *Arthrobacter*, *Marinobacter*, *Neisseria*, *Ralstonia*, *Ochrobactrumle*, *Exiguobacterium*, *Planomicrobium*, *Propionibacterium*, *Kocuria*, *Rhodococcus* and *Stenotrophomonas*	Aromatic and aliphatic hydrocarbons	Ali *et al.* [143]
*Anthocleista*, *Sarcophrynium*, *Canna*, *Colocassia*, *Musa*, *Cola*, *Citrus*, *Mangifera*, *Terminalia* and *Annona*	*Acinetobacter*, *Flavobacterium* and *Micrococcus*	Diesel and kerosene	Ilori *et al.* [144]
American grass and broad beans	*Rhodococcus* and *Pseudomonas*	*n*-Alkanes, phenanthrene, naphthalene, and biphenyl	Sorkhoh *et al.* [145]
Six ornamental plants	*Pseudomonas*, *Microbacterium*, *Rhizobium* and *Deinococcus*	Phenanthrene	Waight *et al.* [146]
*Azalea indica*	*Pseusomonas putida* TVA8	Toluene	De Kempeneer *et al.* [147]
Soybean, clover and *Arabidopsis thaliana*	*Sphingomonas* and *Methylobacterium*	Methanol (via proteomics)	Delmotte *et al.* [122]
Thirteen different plant species from Japan	*Methylomonas*, *Methylosinus* and *Methylocystis*	Methane	Iguchi *et al.* [148]
Four *Prunus* species	*Sphingomonas* and *Methylobacterium*	Methanol (via genomics)	Jo *et al.* [149]
Rice	*Alpha, Beta and Gamma-proteobacteria*, *Actinobacteria*, *Bacteroidetes* and *Firmicutes*	Methanol (via metaproteogenomics)	Knief *et al.* [121]
*Arabidopsis thaliana*	*Hyphomicrobium*	Chloromethane	Nadalig *et al.* [150]
*Phaseolus vulgaris*	*Arthrobacter chlorophenolicus A6*	4-chlorophenol	Scheublin *et al.* [151]
Foliage of an apple orchard	3 *Arthrobacter* sp.	4-chlorophenol	Scheublin and Leveau [152]

## 4. Inorganic Air Pollutants (IAP)

### 4.1. Definition and (Human) Toxicity

The most important and common inorganic air pollutants are SO_2_, CO_2_, CO, NOx and O_3_.

Sulfur dioxide (SO_2_) previously was produced in large amounts during the combustion of coal and other fuels in industrial and domestic use. Nowadays, more low-sulfur-containing fuels are applied for the generation of energy, and SO_2_ concentrations have strongly decreased. As SO_2_ is a stinging gas, it can cause breathing problems. Moreover, SO_2_ is a major component of acid rain [153].

Carbon dioxide (CO_2_) is the major greenhouse gas emitted through anthropogenic activities (mainly the combustion of fossil fuels for energy and transportation). While CO_2_ emissions originate from various natural sources, the increase in emissions in the atmosphere since the industrial revolution is caused by human-related emissions [154]. Carbon dioxide is naturally present in the atmosphere as part of the Earth’s carbon cycle. However, human activities are significantly affecting this carbon cycle in two ways. Anthropogenic emissions on the one hand are an additional supply of CO_2_ in the atmosphere and on the other hand they affect the ability of natural sinks, like forests, to remove CO_2_ from the atmosphere. This increase in CO_2_ concentrations in the atmosphere is strongly contributing to global climate change, including rising surface temperatures, melting ice and snow, rising sea levels, and increasing climate variability. These climate changes are believed to have a significant impact on human health [155].

Oxides of nitrogen (NO_x_) comprise nitric oxide (NO) and nitrogen dioxide (NO_2_). Since NO is a very unstable free radical that is not adsorbed to surfaces in significant amounts [156], of the two NO_x_ forms, NO_2_ is of primary interest for deposition studies. As such, the US Environmental Protection Agency (US EPA) uses NO_2_ levels as an overall indicator of the atmospheric NO_x_ status. The major anthropogenic emission sources for NO_x_ are combustion processes, especially those from automobile traffic [157,158]. At high concentrations, NO_2_ can be toxic to humans [159], but at ambient levels it is expected to pose little risk as such. However, NO_2_ plays a key role in the ozone generating photochemical oxidant cycle, which is of most concern to human health [160].

Ozone (O_3_) is formed in the troposphere when sunlight (more specifically UV-radiation) induces complex photochemical reactions with NO_x_, VOCs and CO. Several public health studies have demonstrated the significant associations between outdoor concentrations of tropospheric ozone and a high variety of adverse outcomes [160,161], including premature mortality, hospital admissions for respiratory disease, urgent care visits, asthma attacks and restrictions in activity [162].

### 4.2. Role of Plants during IAP Phytoremediation

Although inorganic air pollutants cause pernicious effects of varying magnitudes on some plant species, there are also several plant species that are more tolerant and can act as sinks by bioaccumulating the pollutants in their cells and tissues.

For example, in a modeling study by Nowak *et al.* [11], urban trees are shown to remove significant amounts of air pollution thereby improving urban air quality. Total annual air pollution (O_3_, PM_10_, NO_2_, SO_2_, CO) removal by US urban trees was estimated at 711,000 metric tons (3.8 billion dollar value). Moreover, ozone studies that integrate temperature, deposition and emission effects of trees reveal that trees can cause significant reductions in ozone concentrations in urban areas [163,164,165]. Bytnerowicz *et al.* [166] measured differences in O_3_ concentrations between above and below-forest canopies that exceeded 50 ppb, meaning a 40% improvement. In a study in Shanghai, China, SO_2_ and NO_2_ concentrations decreased by 5.3% and 2.6%, respectively, when comparing concentrations in external urban woodland and at a distance of 50–100 m into the forest [46].

SO_2_ mainly enters the leaves through the stomata, following the same diffusion pathway as CO_2_. Once in the leaf cells, it might be detoxified and utilized in a “reductive sulfur cycle” to form sulfur containing amino acids needed for growth and development, as if they had been absorbed through the roots [153]. In this way, if concentrations are not too high, SO_2_ air pollution might provide a sulfur source to the plant. However, in urban areas, these concentrations might be so high that the plant’s detoxification system fails and injury (such as stomatal closure and photosynthesis inhibition) cannot be avoided [153].

Since plants remove vast amounts of CO_2_ from the atmosphere, they are major natural carbon sinks on earth [167]. Mainly through photosynthesis, plants lock up the carbon dioxide from the atmosphere in their own biomass for short and long-term periods (from one year to several hundreds of years in case of some tree species). Although most of the biomass undergoes decomposition and mineralization, a small fraction of it is transformed (also by the microbiome) to humus that is storing CO_2_ for periods of 3000 years and even more [168]. Significant differences are noticed both in CO_2_ uptake by plants as well as in species’ ability to create humus. Those that are effective in both processes shall be identified and incorporated into urban green infrastructure. The process of uptake and long-term storage of atmospheric carbon dioxide is called carbon sequestration [169]. In this sense, carbon sequestration has been proposed as a measure to stop or reverse the increase of CO_2_ in the atmosphere [170]. Although C-sequestration is of high interest in the context of air pollution and climate change, it is such a complex process and going more into detail would not fit within the scope of this review. A recent review on soil organic carbon sequestration is provided by Lorenz and Lal [171].

Several authors have demonstrated the ability of plants to take up atmospheric NO_2_ and incorporate it into different nitrogen pools within the plant [172,173,174], suggesting the possibility for the use of NO_2_ as an alternative fertilizer and in turn the use of plants for air pollution control [175]. Removal of atmospheric NO_2_ by plants occurs via adsorption to the leaf (and root) surface and stomatal uptake to the apoplast [176]. Although some authors have observed high adsorption to leaf surfaces [177,178], stomatal uptake remains the uptake route of primary importance. As NO_x_ is one of the precursors of the photochemical reaction, after entering into the plant, most of them are metabolized to organic compounds (such as amino acids) through the nitrate assimilation pathway. Although NO_2_ might rather act as a nutrient for plants, at higher levels and prolonged exposure, it might become phytotoxic [167].

Plants are able to adsorb ozone by cuticle deposition and to absorb it through stomatal apertures. The first process (adsorption) is only relevant under high surface moisture [179] while the stomatal absorption is the major contributor to the total uptake of ozone [180]. Ozone disappears when reacting in the gas phase or when making contact with cuticles and apoplastic compounds. At the cuticle level, ozone can react with a multitude of waxes, salts, ions, biogenic and anthropogenic VOC and many other compounds, especially in conditions of wetness [179,181]. The fate of ozone after entering the stomata is not fully understood. Most probably, ozone indirectly affects the denaturation of membrane lipids [182] rapidly reacting with all compounds in the apoplast and in the gas phase, and generating reactive oxygen species (ROS) [183]. Chronic stresses with exposure to moderate ozone concentrations usually produce biochemical and physiological changes [184,185,186]. Exposure to acute tropospheric ozone levels leads to visible injuries [187].

### 4.3. Role of Plant-Associated Microorganisms during IAP Phytoremediation

Concerning inorganic air pollution, the knowledge that is available about the role of the plant-associated microbiome during phytoremediation is very limited.

Considering the nitrogen and sulfur metabolisms that exist for microorganisms, we might expect (at least part of) the plant-associated microbiome to be involved in NO_x_ and SO_2_ capturing. Only Papen *et al.* [188] demonstrated that chemolithoautotrophic bacteria might contribute to the large NO_2_ deposition rates on leaves. In case of CO_2_, autotrophic microorganisms using CO_2_ as carbon source are expected to be of interest.

Moreover, in the context of carbon sequestration, it is known that the plant’s microbiome affects humus formation and composition [189]. Until recently, the potential contribution of mycorrhizal fungi to carbon sequestration in soil organic matter (SOM) was largely overlooked [189]. Clemmens *et al.* [190,191] demonstrated the significance of mycorrhizal input by showing that the majority of C stored in SOM in a boreal forest system originated from roots and fungi. From the other point of view, Lesaulnier *et al.* [192] showed that the elevated CO_2_ concentrations in the atmosphere significantly affect soil microbial diversity associated with aspen.

Ozone is known as an antimicrobial agent. Therefore, the contribution of the microbiome during ozone phytoremediation will probably be limited to toxicity abatement. As ozone is known to generate ROS, bacteria with high antioxidative properties [74,75] can play a role in ROS detoxification.

In general, all plant growth promoting traits of the plant-associated microbiome might benefit plant growth and development upon exposure to inorganic air pollutants.

## 5. State of the Art and Future Challenges

Reducing air pollution is much more of a challenge than control of soil and water pollution, and to meet this demand, new innovative ideas and methods are required. In plants, together with their microbiomes, lies huge unexploited potential for purifying both indoor and outdoor air. In general, the average percent air quality improvement (only taking into account O_3_, PM_10_, NO_2_, SO_2_, CO) due to plants is estimated to be relatively low (around 1%) [11]. However, the improvement counts for multiple pollutants and the actual magnitude of pollution removal can be significant [11].

Moreover, plants together with their microbiomes in urban green infrastructures provide a wide variety of ecosystem services that help to combat many urban ills and improve life of citizens [193].

From the above it is clear that plants and their associated microorganisms are very promising as a tool to improve air quality and in these plant-microbe systems, both partners are of high importance. Moreover, in previous research it became clear that, in case of soil and/or groundwater pollution, the efficiency of phytoremediation can be strongly improved by the further exploitation of plant–microbe interactions [16,194,195,196,197]. Plant-associated bacteria with the desired characteristics were exploited by enriching them in plants by means of inoculation. After inoculation, an increased biomass, contaminant uptake and/or degradation as well as a reduced phytotoxicity could be achieved [126,127,198,199].

Similarly, in the framework of phytoremediation of air pollution, a future challenge might be to select the most promising plant species naturally accompanied with specific microbial communities (with respect to adsorption, uptake, degradation, detoxification and BVOC emission capacity). The exposure of plants to local conditions and pollutants plays an important role in the ecology of phyllobacteria. However, it turns out that plant species are often accompanied by the same bacterial species even if they grow on another continent [200]. Based on next generation sequencing research, it is clear that the taxonomic composition of the rhizosphere, root-endosphere and other plant-endophytic bacterial communities is different from the bulk soil. It is suggested that this occurs in a two-selection step, in which plant rhizodeposits mediate a substrate-driven community shift in the rhizosphere, and the host–genotype innate immune system fine-tunes the microbial profile in the selection of root endophyte assemblages [13,201,202]. Particularly with respect to the long-term effectiveness of phytoremediation, the role of the rhizosphere as a resource for specific microbial strains as well as their conservation under environmental pollution might be of high importance.

Once plant species with naturally associating microorganisms are selected, the next step will be their enrichment with the most promising microbes (with respect to degradation, transformation, sequestration, detoxification and plant growth promotion capacity) in order to obtain the best performing bioaugmented plant–microbe systems. As the phyllosphere is scavenging the major part of the air pollutants, in this case, phyllosphere is recommended instead of (or next to) rhizosphere inoculation. To the best of our knowledge, phyllosphere inoculation and its effect on phytoremediation efficiency is only described by De Kempeneer *et al.* [147]. In their work, the *Azalea indica* phyllosphere was inoculated with a toluene degrading culture of *Pseudomonas putida* TVA8. Plants were exposed to toluene, and in comparison with non-inoculated control plants, the toluene removal rate was significantly increased after phyllosphere inoculation.

Moreover, plants with their associated microorganisms play a leading role in maintaining biodiversity and ecological sustainability of urban green infrastructures, and basic knowledge of this symbiosis is of high importance for human health and environmental sustainability. Air pollution affects ecosystems in a number of ways, and impacts should be quantified across a range of ecosystem service types, to provide a more holistic view of the effects.

Clearly, the removal of air pollutants (climate mitigation) results in health benefits. The adequate planning of green areas has a substantial positive influence on health of urban dwellers in the long term. In cities, the use of plants moreover improves the microclimate [203] and reduces negative side effects of climate change (climate adaptation) in multiple ways by: blocking unwanted sun radiation during summer resulting in lower building warming up, releasing moisture to the surrounding atmosphere by evapotranspiration resulting in lower temperatures (especially with regards to the heat island effect), and reducing wind speed by functioning as a wind buffer leading to a reduction in heat losses in winter. In addition, plants can also be exploited to intensively reduce carbon footprint by absorbing CO_2_ and (in an optimal design) even realize extremely long term carbon sequestration [204,205].

Moreover, the use of plants has additional benefits for humans (ecosystem services) compared to conventional technologies that have been rather well-documented. Biodiversity can be improved by the presence of green infrastructure within a city with a relevant connection function with the surrounding area [206]. In cities, characterized by a high density of habitation and activity, noise is perceived as a main disruption. Vegetation acts as a natural noise buffer [207]. In case of rainfall, large hardened sections make the city entirely dependent on the drainage system for the discharge of the storm water, which often results in local flooding. Green urban infrastructure can collect and temporarily retain these sudden floods, allowing the discharge peak to flatten [208,209]. Besides the direct health effects which result from air quality and local climate improvement, the presence of structural green in the city also has other health effects by the mere sight of nature, being in a natural environment, and the potential to be physically active [210]. More specifically, in addition to purifying the air, green infrastructure will also make daily activities such as walking and cycling more attractive for commuting to school, work and services [211].

From the above, it is clear that plant-based technologies can positively affect ecosystems in many ways. Further, we have to take into account that the fitness and expression of key plant traits important for phytoremediation (e.g., root architecture, above-ground biomass, leaf area/number) in any environment (natural or altered) are driven by below-/above-ground multi-trophic interactions [212,213,214]. Therefore, a sustainable phytoremediation of contaminated ecosystems can only be obtained when these complex interactions are taken into consideration. Phytoremediation represents an integrated approach to combat air pollution and climate change and, at the same time, safeguard or improve other aspects of human well-being. These findings therefore suggest that plant-based technologies should be a crucial part of a holistic strategy to achieve the worldwide objectives regarding clean air and enhanced human well-being.
